# A Comparative Assessment of the Influences of Human Impacts on Soil Cd Concentrations Based on Stepwise Linear Regression, Classification and Regression Tree, and Random Forest Models

**DOI:** 10.1371/journal.pone.0151131

**Published:** 2016-03-10

**Authors:** Lefeng Qiu, Kai Wang, Wenli Long, Ke Wang, Wei Hu, Gabriel S. Amable

**Affiliations:** 1 Institute of Rural Development, Zhejiang Academy of Agricultural Sciences, Hangzhou, China; 2 School of Marine Sciences, Ningbo University, Ningbo, China; 3 Institute of Digital Agriculture, Zhejiang Academy of Agricultural Sciences, Hangzhou, China; 4 Institute of Remote Sensing and Information System Application, Zhejiang University, Hangzhou, China; 5 Department of Geography, University of Cambridge, Cambridge, United Kingdom; University of Vigo, SPAIN

## Abstract

Soil cadmium (Cd) contamination has attracted a great deal of attention because of its detrimental effects on animals and humans. This study aimed to develop and compare the performances of stepwise linear regression (SLR), classification and regression tree (CART) and random forest (RF) models in the prediction and mapping of the spatial distribution of soil Cd and to identify likely sources of Cd accumulation in Fuyang County, eastern China. Soil Cd data from 276 topsoil (0–20 cm) samples were collected and randomly divided into calibration (222 samples) and validation datasets (54 samples). Auxiliary data, including detailed land use information, soil organic matter, soil pH, and topographic data, were incorporated into the models to simulate the soil Cd concentrations and further identify the main factors influencing soil Cd variation. The predictive models for soil Cd concentration exhibited acceptable overall accuracies (72.22% for SLR, 70.37% for CART, and 75.93% for RF). The SLR model exhibited the largest predicted deviation, with a mean error (ME) of 0.074 mg/kg, a mean absolute error (MAE) of 0.160 mg/kg, and a root mean squared error (RMSE) of 0.274 mg/kg, and the RF model produced the results closest to the observed values, with an ME of 0.002 mg/kg, an MAE of 0.132 mg/kg, and an RMSE of 0.198 mg/kg. The RF model also exhibited the greatest R^2^ value (0.772). The CART model predictions closely followed, with ME, MAE, RMSE, and R^2^ values of 0.013 mg/kg, 0.154 mg/kg, 0.230 mg/kg and 0.644, respectively. The three prediction maps generally exhibited similar and realistic spatial patterns of soil Cd contamination. The heavily Cd-affected areas were primarily located in the alluvial valley plain of the Fuchun River and its tributaries because of the dramatic industrialization and urbanization processes that have occurred there. The most important variable for explaining high levels of soil Cd accumulation was the presence of metal smelting industries. The good performance of the RF model was attributable to its ability to handle the non-linear and hierarchical relationships between soil Cd and environmental variables. These results confirm that the RF approach is promising for the prediction and spatial distribution mapping of soil Cd at the regional scale.

## Introduction

Cadmium (Cd) is a toxic metal element that causes extensive concern because of its extremely harmful effects on animals and humans [1]. Due to the low permissible exposure limit of Cd, overexposure may occur even in situations in which trace quantities of Cd are found and can result in metal fume fever, chemical pneumonitis, pulmonary edema, and death [2]. Cd accumulation through the food chain is also harmful to animal and human health. Especially in southern China and northeastern Vietnam, the problem of human exposure to Cd via rice (*Oryza sativa*) intake is of increasing concern [3,4]. The natural concentration of Cd in soils is relatively low; Cd comprises only approximately 0.1 mg/kg of the Earth’s crust, and its concentration mainly depends on the geochemistry of the parent material [5,6]. Consequently, soil Cd contamination primarily results from a variety of human activities. Mining [7], smelting [8], electroplating, and scrap metal recycling are coincident with the most important sources of environmental pollution by metals and metalloids [9,10]. Therefore, the challenge is to understand the spatial distributions and high local variabilities in soil Cd concentrations that are caused by the influences of human activities. Such understanding is needed for the preparatory work for remediation. Thus, the evaluation and application of deterministic environmental factors to model the spatial distributions of soil properties (including soil Cd) has been proposed to be an efficient methodology that can serve as an alternative solution to expensive and waste of time soil sampling [11–14].

Numerous statistical techniques for estimating the spatial distributions of soil properties at different scales have been developed and tested within a digital soil-mapping (DSM) framework [15]. In these techniques, linear regression is one of the most frequently used model because of its simplicity, efficiency, and straightforward interpretation [16]. Linear regression models assume that the relationships between the predictor variables and response variables are linear. However, the relationships between soil properties and environmental parameters are often complex and non-linear due to the influences of many factors, such as climate, parent material, topography, and human activities [17]. Machine-learning techniques, such as classification and regression tree (CART) analysis, have been proposed to overcome the shortcomings of linear regression models and to account for the non-linear relationships between soil properties and environmental parameters. CART models can use a wide range of data types and improve the prediction accuracies of spatial models [18,19]. Compared with CART, random forest (RF) modeling, which was developed from CART, is more robust, more resistant to overfitting, and less sensitive to noise in the data [20].

All of these predictive models require an understanding of the factors that control the distributions of the predicted soil properties. Several studies have focused on land use and Cd accumulation in the soil [21–25] because land use data are readily available and extensive and generally represent the impacts of human activities on the soil environment. However, due to lack of detailed description of human activities on the land, land use variables do not always have the ability to play a role in soil Cd prediction [26,27]. When land use variables are employed, significant differences in soil Cd concentrations according to different land use types, for example, woodlands, paddy fields, orchards, vegetable fields, and industrial areas, are expected. However, the impacts of human activities are too complex to be represented by such coarse land use categories, particularly in the rapidly developing area of eastern China. More detailed information regarding land use, including industrial components, factory distributions, transportation, and urbanization, should be incorporated to predict the spatial distributions of soil Cd concentrations [17].

In the present paper, we tested three empirically based models, i.e., a stepwise linear regression (SLR) traditional linear regression model and two machine learning tools, CART and RF, in the prediction and mapping of the spatial distribution of the soil Cd concentration in Fuyang County. The objectives of this study were to develop and compare the performances of the three models in the estimation of the soil Cd content using factors that likely influence soil Cd concentrations and to identify the likely sources of this contamination.

## Materials and Methods

### Ethics statement

All research involved in this study complied with the laws of the People’s Republic of China, and permission for the field experiments in Fuyang County was obtained from the Agricultural Bureau of Fuyang County. The study site did not involve endangered or protected species.

### Site description

**Fig 1:**
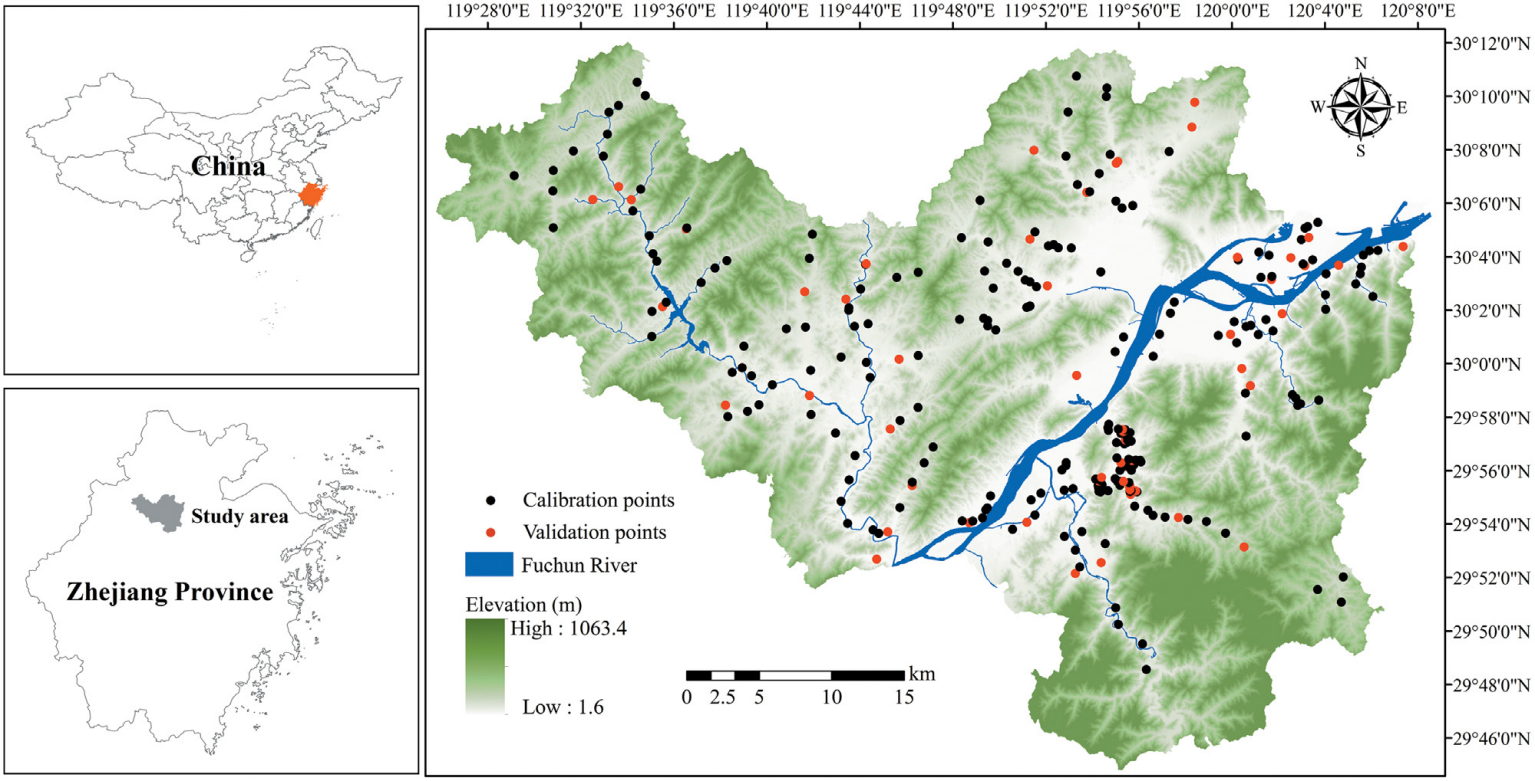
Study area location and sampling points.

The study site was located in Fuyang County in northern Zhejiang Province, which is one of the most economically developed provinces in China (Fig 1). This county (119°25′00″-120°19′30″ E, 29°44′45″-30°11′58.5″ N) has an area of 1,831 km^2^ and a landscape characterized by a mountain and valley topography. The elevation ranges from 1.6 to 1,063.4 m. In recent decades, urbanization and industrialization have occurred at an unprecedented pace in Fuyang County. In the study area, paper mills, metal smelters, hardware machinery factories, and building material factories have been extensively developed. Moreover, the total number of motor vehicles has rapidly increased, and heavy traffic exists throughout the study area [28].

### Data collection

Soil samples (n = 276) were collected from the agricultural land across the study area in 2005 based on the uniformity of plot distributions and the land use types in the study area (Fig 1). Each of the samples were collected at a depth of 0–20 cm from five sampling points within 5 m around a specific sampling location and then mixed. A global positioning system (GPS) was used to precisely locate every sampling location. All samples were air dried at room temperature. Stones and plant residuals in the soil samples were manually removed, and the soil samples were then ground to pass through a 2 mm sieve. These samples were analyzed for soil organic matter (SOM), pH, and Cd. The soil pH was determined with a pH meter with a soil/water ratio of 1:2.5, and the SOM was determined using the K_2_Cr_2_O_7_-H_2_SO_4_ oxidation method. Total Cd was determined by digesting the soil sample with a mixture of nitric acid (HNO_3_) and perchloric acid (HClO_4_) followed by measurements, which were determined by inductively coupled plasma mass spectrometry (Agilent 7500a, USA) [29]. The accuracy of determinations was checked using national standard product (GBW 07401). The quality control gave good precision (S.D. < 10%) for all samples. Detection limit for total soil Cd concentrations was 0.02 mg/kg.

**Fig 2:**
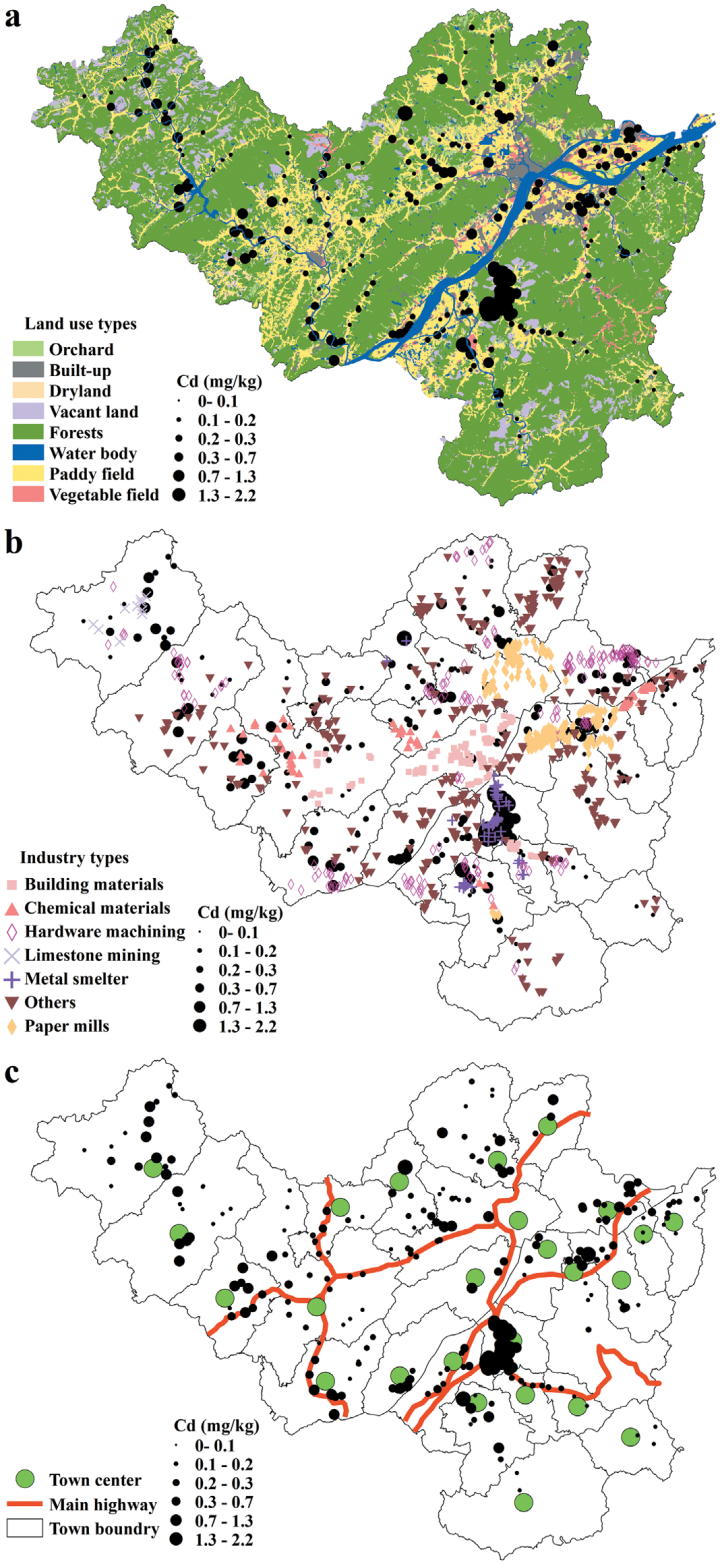
Spatial distribution of the soil Cd concentrations in relation to the (a) land use types, (b) industry types and (c) town center and main highway.

A digital elevation model (DEM) with a 25 m spatial resolution and land use data including information on the land use types (Fig 2a), industry types (Fig 2b), and town center and main highway (Fig 2c) for the year 2005 were obtained from the Bureau of Land and Resources of Fuyang County.

### Model construction

The sampling locations were added to an ArcGIS 10.0 (ESRI Inc., Redlands, CA, USA) geodatabase in which the soil properties were associated with the sampling points. The point location layer was intersected with the land use data and the DEM to obtain the independent variables, and the soil Cd concentrations were the dependent variables. Based on a review of the literature [14,17,30–34], we selected a priori 16 environmental variables (Table 1) which represent soil properties, topographic, and anthropogenic activities to explain the spatial variability in the soil Cd concentration.

**Table 1:** The environmental variables selected for model calibration.

Environmental variable	Abbreviation	Unit	Type	Mean	Minimum	Maximum
**(a) Soil data**						
**pH**	pH	-	Continuous	6.06	4.40	8.31
**Soil organic matter**	SOM	%	Continuous	3.12	0.57	6.50
**(b) Elevation**	ELE	m	Continuous	37.66	4.43	146.26
**(c) Land use types**						
**Vegetable field** ^a^	Lu_Veg	-	Binary	-	-	-
**Paddy field** ^a^	Lu_Pad	-	Binary	-	-	-
**Dryland** ^a^	Lu_Dry	-	Binary	-	-	-
**Forests** ^a^	Lu_For	-	Binary	-	-	-
**Orchard** ^a^	Lu_Orc	-	Binary	-	-	-
**(d) Distance to industry**						
**Metal smelter**	Dmetal	km	Continuous	9.07	0.05	30.69
**Hardware machining**	Dhardware	km	Continuous	3.39	0.04	11.38
**Building materials**	Dbuild	km	Continuous	7.43	0.03	26.91
**Chemical materials**	Dchemical	km	Continuous	6.24	0.06	18.50
**Paper mills**	Dpaper	km	Continuous	10.73	0.04	40.58
**Other industry**	Dother	km	Continuous	34.27	0.34	59.43
**(e) Distance to highway**	Droad	km	Continuous	3.10	0.002	21.97
**(f) Distance to town center**	Dtown	km	Continuous	3.14	0.33	8.81

^a^ Binary variable (0 for absence and 1 for presence).

Pioneering research on soil Cu, Zn, Pb, and Cd contamination in Fuyang County by Zhang et al. (2008 and 2009) [17,28] demonstrated that Fuyang’s soil was elevated in Cu, Zn, Pb, and Cd in the areas where industrial plants, towns, and roadways were concentrated. Thus, in our study, the distances to different kinds of industry plants, highways, and town centers were used as important predictors of soil Cd concentrations. Besides, it is reported that application of manure, fertilizers, pesticides, and herbicides were closely linked with soil Cd accumulation [17], therefore agricultural land uses were also selected in analysis. As a result, five main land use categories were classified in the study area. These were vegetable field, paddy field, dryland, forests and orchard. Again, soil properties and topography are two key factors determining natural contents and transport processes of soil Cd. Nevertheless, the measured Cd concentrations were ranged from 0.006 to 2.216 mg/kg with the mean values of 0.322 ± 0.394 mg/kg in the study area. 68.8% of the measured values exceed their according background values of 0.14 mg/kg for Cd in soil at Zhejiang Province and 30.1% of the samples are higher than the Chinese Environmental Quality Secondary Standard for Soils of 0.3 mg/kg (GB 15618–1995) [24]. Highly elevated Cd concentrations coupled with its high spatial variability suggest that anthropogenic inputs may be the primary source of Cd in the study area. This has indicated that anthropogenic factors are more likely to predict soil Cd concentrations than natural factors. Thus, we only selected pH, SOM, and elevation in analysis as representative factors of natural environment. Moreover, soil pH and organic matter influence soil Cd concentrations because they are strongly correlated with the solubility and mobility of soil Cd. And elevation is usually the most readily available among topographic variables. In this study, soil pH were ranged from 4.40 to 8.31, with the mean value of 6.06; SOM ranged from 0.57 to 6.50%, with the mean value of 3.12%; and elevation ranged from 4.43 to 146.26 m, with the mean value of 37.66 m (data in S1 Dataset).

#### Stepwise linear regression

Following a method developed by Montgomery [35], we constructed a SLR model that predicted soil Cd concentrations in the study area with environmental features. Because the original data failed the Kolmogorov-Smirnov normality test (*P* < 0.05), they were log transformed to ensure a normal distribution. Then, the construction of SLR model was conducted in SPSS^®^ (Version 16.0) software.

Following the removal of the variables that were not statistically significant, the SLR parameter estimates were conducted again. ELE, pH, Dmetal, and Dtown were retained in the model. To apply the estimate parameters across the study area, raster data layers for all of the predictor variables (i.e., ELE, pH, Dmetal, and Dtown) were created. Within ArcGIS, the soil pH distribution was interpolated by ordinary kriging, which is a commonly used interpolation method. Raster datasets were created for Dmetal and Dtown by the Euclidean Distance tool in ArcGIS. To match the spatial resolution of the DEM used in the study, a 25 m^2^ cell size was used in the construction of all raster layers. Additionally, only areas described as agricultural land were retained in the dataset. Areas described as built-up lands and water bodies were removed from the dataset due to no soil was present there.

**Fig 3:**
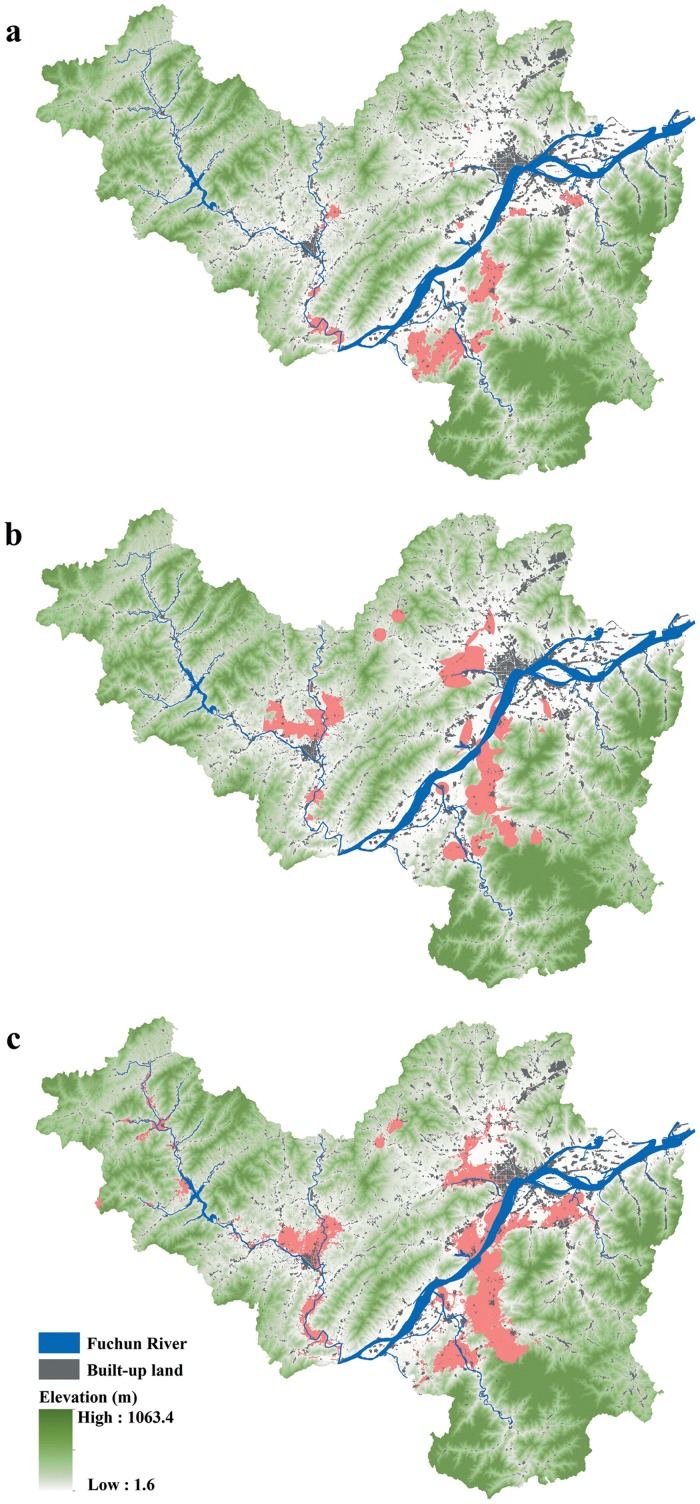
(a) SLR, (b) CART, and (c) RF predictions of Cd in agricultural soils in Fuyang County. The areas in red are predicted to exceed the Chinese soil Cd guide limit of 0.3 mg/kg.

Based on the parameters that resulted from the SLR, the raster calculator tool in ArcGIS was used to predict the distribution of soil Cd concentrations. The resulting raster dataset reflected the spatially distribution of log-transformed Cd concentration values. Finally, the resulting raster dataset was reclassified in ArcGIS such that the cells reflected a binary dataset of values that exceeded or below the Chinese soil Cd guide limit of 0.3 mg/kg (Fig 3a). The construction of the binary dataset was conducted for two reasons: (1) to facilitate the follow-up management and (2) for comparison with the CART model, which separates the Cd values into classes.

#### Classification and regression tree

Classification and regression tree (CART) is a machine-learning algorithm that can be used to split a complex decision into several branched and simplified decisions and may lead to an easier solution [36]. The trees are grown by recursively partitioning a dataset of the dependent variable into a series of binary subsets. Compared to SLR and other traditional general linear models, one potential advantage of CART is the ability to discover unexpected and fresh patterns in non-normal and complex data.

In the CART model, the Cd concentration for each sample was categorized as either low (0–0.3 mg/kg) or high (> 0.3 mg/kg) and used as a target variable, and the 16 environmental variables were utilized as model inputs. The CART analysis was conducted with the R statistical software using the rpart package [37]. Next, the prediction rules calculated by the CART model were translated into a series of branched and simplified statements that were constructed using the raster calculator tool in ArcGIS. The resultant map revealed the areas in which the soil Cd was predicted to exceed the Chinese soil Cd guide limit of 0.3 mg/kg (Fig 3b).

#### Random forests

Developed from CART analysis, which produces a single tree, random forests (RF) combine a forest of uncorrelated trees created with the CART procedure [38]. Each tree is constructed by a randomly selected subset of training data. The remaining training data, which are called “out-of-bag”, are used to estimate prediction error and variable importance [39]. Three training parameters, i.e., (i) the number of trees to grow (*n*_tree_), (ii) the number of predictor variables used to split each node (*m*_try_), and (iii) the minimum number of observations at the terminal nodes of the trees (*nodesize*), were set to 1,000, 12, and 5, respectively.

We used the randomForest package in the R environment to create an RF model based on the 222-sample training dataset [20]. Next, the model was applied to a continuous ASCII dataset that contained the same independent variables used to construct the RF model. The output result from the randomForest package was an ASCII format file and then was converted to a raster dataset using ArcGIS. Finally, the raster dataset was reclassified to a binary map that also displayed the areas in which the soil Cd was predicted to exceed the Chinese soil Cd guide limit of 0.3 mg/kg (Fig 3c).

### Validation

To evaluate the model performances, 54 sampling points (Fig 1) were randomly selected from the original 276 soil samples as the validation samples using the subset features tool of the Geostatistical Analyst extension in ArcGIS. The accuracy assessment was based on analysis of the error matrix, which was a square array of dimensions *n* × *n* (*n* was the number of classes). This matrix revealed the relationship between the estimated and measured Cd concentrations. The total accuracy and the kappa coefficient were selected to evaluate the prediction accuracy [17]. The former is the ratio of the total number of correctly predicted Cd concentrations to the total number of validation samples (n = 54), and the latter uses all of the information in the error matrix and ranges from 0 to 1. A value of 1 implies perfect agreement, and values below 1 imply less than perfect agreements. Fleiss [40] characterized kappa coefficients below 0.40 as poor, 0.40 to 0.75 as fair to good, and over 0.75 as excellent. Additionally, the mean error (ME), root mean squared error (RMSE), mean absolute error (MAE), and coefficient of determination (R^2^) were calculated to assess the accuracy of the predicted Cd concentrations [11,41].

## Results and Discussion

### Relative importance of the predictor variables

The estimated ELE, pH, and Dmetal parameters were all highly significant at the *P* < 0.001 level, and Dtown was significant at *P* < 0.05 (Table 2). The constant of the model was also significant at the *P* < 0.001 level. These results indicated that ELE, pH, Dmetal, and Dtown substantially influenced the spatial distribution of Cd concentrations.

**Table 2:** Estimated parameters of the SLR model.

Variable	Parameter	Std. error	t value	p-value
**Constant**	-3.619	0.379	-9.541	0.000
**ELE**	-0.007	0.002	-4.314	0.000
**pH**	0.473	0.061	7.79	0.000
**Dmetal**	-0.038	0.008	-5.012	0.000
**Dtown**	-0.087	0.035	-2.503	0.013

**Fig 4:**
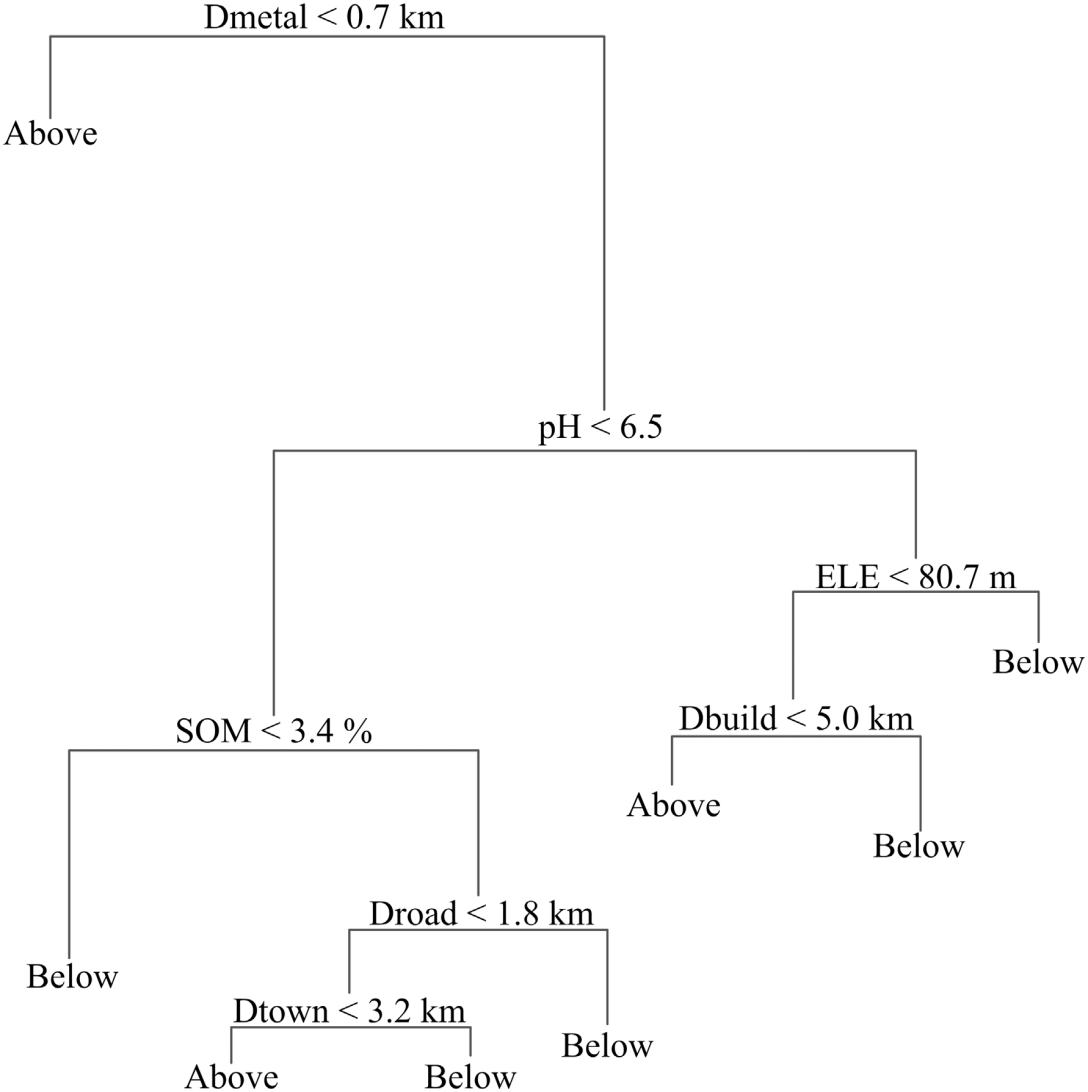
CART model developed to predict Cd in agricultural soils in Fuyang County. The lengths of the lines or "branches" are proportional to the variance explained, and longer branches explain more variance. Below: 0–0.3 mg/kg; Above: > 0.3 mg/kg.

The simulated tree model contained 8 terminal nodes and 7 independent variables, including Dmetal, pH, SOM, ELE, Dbuild, Dtown, and Droad. Dmetal was the most important factor for classifying the statuses of Cd contamination (Fig 4). Samples located less than 0.7 km from any metal smelter that influenced soil Cd concentration were predicted to contain Cd concentrations that exceeded the Chinese soil Cd guide limit of 0.3 mg/kg.

The variations in the misclassification error and the numbers of terminal nodes as the predictors were excluded one by one from the constructed CART models and are listed in Table 3. The numbers of terminal nodes for the 7 tree models were within the range of 7 to 9, which indicates that these similarly complicated tree models did not grow too tall or overly complex. Based on the magnitude of the increase in the misclassification error, Dmetal was the most important variable for explaining the spatial variations in the Cd concentrations. This finding was unsurprising for statistical and theoretical reasons. Statistically, the Pearson correlation identified between the Cd concentrations and Dmetal values was significant (*P* < 0.01), which indicated that Dmetal was a good predictor of Cd concentration. Theoretically, this high correlation can be ascribed to the strong relationship between soil Cd accumulation and metal smelting industries. It is reported that nonferrous metal industry is strongly correlated with soil Cd contamination throughout the world [42–45], and such metal smelting is one of the pillar industries in Fuyang County. Large quantities of Cd were released into the surrounding environment due to intense emissions of smelting gases and improper disposal of solid wastes [46]. The contributions of the remaining predictors to the models were more or less the same because their exclusions marginally increased the misclassification error. These findings indicate that although these variables influenced soil Cd accumulation, they did not exhibit significant effects on Cd accumulation compared with Dmetal.

**Table 3:** The relative importance of the variables for explaining soil Cd variation as indicated by variations in misclassification errors and the numbers of terminal nodes in the CART model following the exclusion of predictors.

Variable	Misclassification error rate	Number of terminal nodes
**Missing Dbuild**	8.11%	9
**Missing ELE**	9.01%	9
**Missing Dtown**	9.91%	8
**Missing SOM**	10.36%	7
**Missing Droad**	10.36%	8
**Missing pH**	10.81%	7
**Missing Dmetal**	13.51%	8

It was difficult to judge the importance of each predictor in the RF model because RF algorithms do not reveal the functional relationships between the target and predictor variables. Due to this limitation in interpretability, RF models are called “black box” approaches [41].

### Spatial prediction and Cd concentration mapping

The predictions of the Cd levels in the agricultural soils based on the SLR, CART, and RF models are displayed in Fig 3. Generally, the three prediction maps were similar and realistic in terms of the spatial patterns of Cd contamination. The areas in which the soil Cd concentration exceeded 0.3 mg/kg were primarily located in the alluvial valley plain of Fuchun River and its tributaries. This finding clearly reflected the effects of industrial operations, especially metal smelting activities, on soil Cd accumulation because these areas were industrially well developed according to the statistical data (Fig 2b). Moreover, the most dramatic urban sprawl occurred in these regions (Fig 2c). Rapid urban expansion also explained the high Cd concentrations [33] in these areas. In contrast, the areas in which the soil Cd concentration was below 0.3 mg/kg were distributed in the higher-altitude areas, and this pattern could be partly explained by presence of indigenous forests that are hundreds of years old in these areas.

The area predicted to exceed the Chinese soil Cd guide limit of 0.3 mg/kg varied among the models. The SLR model predicted the smallest area (32.69 km^2^ or 1.8% of the total modeled area) that would exhibit a soil Cd concentration above 0.3 mg/kg, whereas the RF model predicted the greatest area (89.30 km^2^ or 4.9%), and the CART model predicted a medium area (68.39 km^2^ or 3.8%) that would exceed 0.3 mg/kg.

The CART model predicted a contaminated area of more than twice the size of that predicted by the SLR. This difference may have resulted from the application of a rule in the CART model that states that the soil Cd will be above 0.3 mg/kg within 0.7 km of any metal smelter regardless of the other environmental factors. This rule is consistent with the observations from the sampled data in that elevated levels of Cd were found next to metal smelters (Fig 2b) and also with other studies that have documented serious risks of Cu pollution within 1,500 m of metal smelters [17]. The RF model predicted an even larger area of contamination than the CART model, which may be attributable to the RF model’s advantage in handling complex data relationships [11]. In the present study, the relationships between soil Cd accumulation and the influences of human activities were nonlinear and hierarchical and were revealed by the SLR and CART models. In contrast to the SLR model, the RF model required no assumptions regarding the relationships between soil Cd concentration and influencing factors and handled the nonlinear and hierarchical relationships without such assumptions.

### Performances of the three models

The validation dataset (n = 54) was used to test the performances of three models. For the SLR, 39 of the 54 validation samples were correctly classified, which resulted in a total accuracy of 72.22% (Table 4). The kappa coefficient, which represents the inter-rater agreement for qualitative (categorical) items, was 0.4048, which indicated a fair to good prediction accuracy from the SLR. Regarding the CART, 38 of the 54 validation samples were correctly classified, which resulted in a total accuracy of 70.37% (Table 4). The kappa coefficient was 0.3949, which indicated a very close to good prediction accuracy of the CART. The RF model correctly classified 41 of the 54 validation samples, which resulted in an overall accuracy of 75.93% (Table 4). The kappa coefficient was 0.5050, which indicated the prediction accuracy of the RF model was the greatest among the three models.

**Table 4:** Error matrices for the SLR, CART and RF predictions of the soil Cd concentrations.

	SLR		CART		RF	
	Low	High	Low	High	Low	High
**Low**	27	5	23	9	25	7
**High**	10	12	7	15	6	16
**Accuracy (%)**	72.97	70.59	76.67	62.50	80.65	69.57

SLR: total accuracy: 72.22% and kappa coefficient: 0.4048. CART: total accuracy: 70.37% and kappa coefficient: 0.3949. RF: 75.93% and kappa coefficient: 0.5050. Low: 0–0.3 mg/kg; High: > 0.3 mg/kg

**Fig 5:**
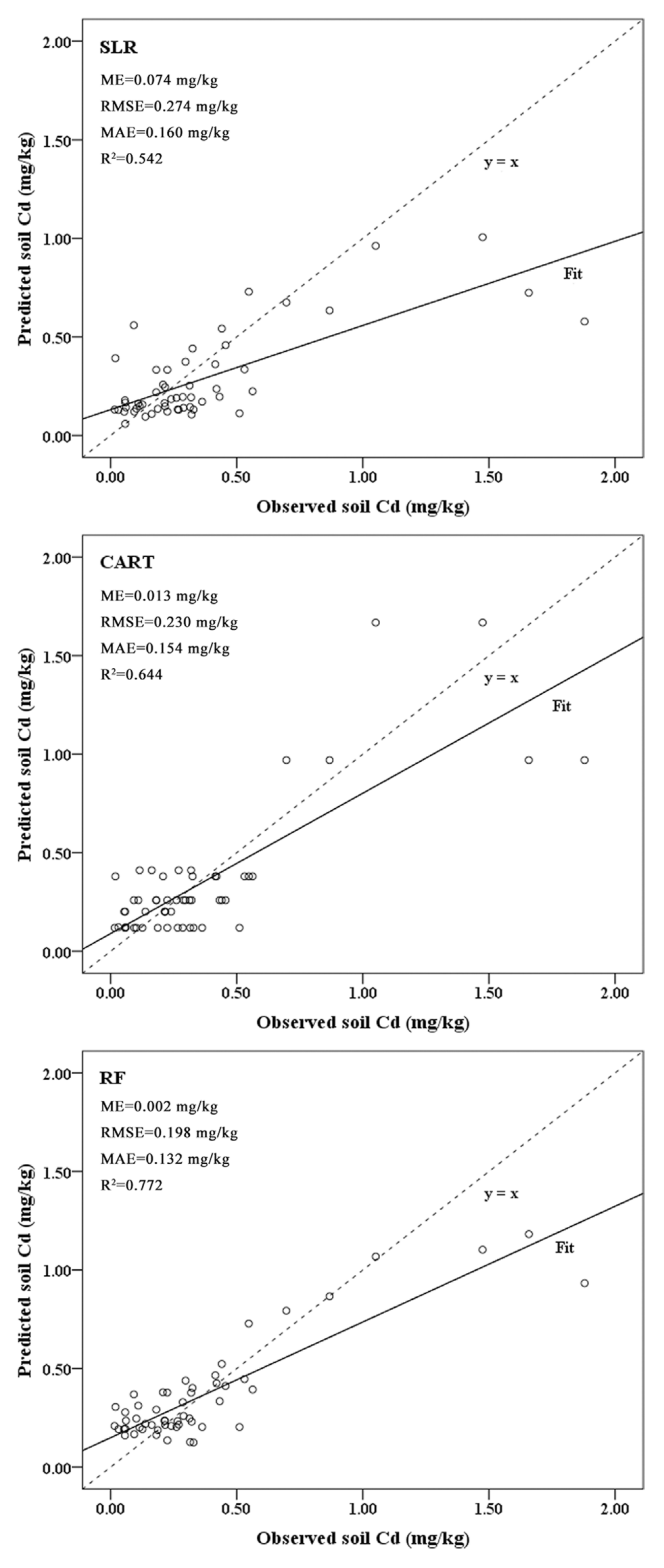
Performances of the SLR, CART, and RF models in the prediction of soil Cd concentrations.

Fig 5 compares the observed and predicted soil Cd concentrations using the validation dataset. This figure also displays the prediction error indices derived from the independent validations of the soil Cd concentrations using the validation dataset. Positive ME values indicate that the models underestimated the Cd concentration. Specifically, the SLR model exhibited the greatest tendency for underestimation, with an ME of 0.074 mg/kg, whereas the RF model exhibited the lowest tendency for underestimation, with an ME of 0.002 mg/kg (Fig 5). Statistically, the MAE is a quantity used to measure the differences between predictions and eventual outcomes. The SLR model exhibited the largest predicted deviation, with an MAE of 0.160 mg/kg, whereas the predictions of the RF model (MAE 0.132 mg/kg) were closest to observed values (Fig 5). Moreover, the RF model exhibited the lowest RMSE (0.198 mg/kg) and the highest R^2^ value (0.772). Compared with other studies [11,41,47], the R^2^ value of the RF model in the present study was slightly higher. This difference may have resulted from differences in the study areas, topographies, sampling strategies, or quantities and qualities of the utilized environmental variables. Hence, the RF method was optimal for predicting the Cd concentrations of the unvisited locations in this context, followed closely by the CART model, which produced predictions with ME, MAE, RMSE, and R^2^ values of 0.013 mg/kg, 0.154 mg/kg, 0.230 mg/kg and 0.644, respectively. The SLR model produced the poorest prediction results, as indicated by the highest values for these three error indices (ME = 0.074 mg/kg, MAE = 0.160 mg/kg, and RMSE = 0.274 mg/kg) and the lowest R^2^ (0.542) in the three models (Fig 5).

The models described above are most likely applicable to other county-scale regions, such as counties in southeastern China with similar urbanization and industrialization processes, although the accuracies of the models may depend on regional characteristics. The SLR model provides a convenient and reasonable method when data sources are limited. The RF model predicted the largest contaminated area. It may be less likely to exclude possible Cd contamination and more protective of environment. However, when working with environmental managing departments, the CART is the preferred method due both to its high accuracy and a series of statements that predict pollution classes which are convenient for translation into public policy.

## Conclusions

In the present study, the RF method was found to be the best method for spatially predicting and mapping soil Cd concentration patterns in Fuyang County, China. The accuracy of the RF model was satisfactory, and the prediction map produced by the RF method revealed a realistic spatial pattern of soil Cd contamination. Compared to the SLR, the RF model performed much better in predicting and mapping the spatial distribution of soil Cd because the RF model proficiently handled the nonlinear and hierarchical relationships between the soil Cd and the main influencing factors. This result confirmed the reliability of the use of RF to model and predict the spatial distribution of soil Cd using environmental variables. This approach could be selected as an alternative methodology to reduce the cost of intensive soil sampling.

Furthermore, analysis of the importance of each variable identified the presence of metal smelting industries as the most important variable for explaining high soil Cd accumulation in the study area. Intense emissions of smelting gases and improper disposal of solid wastes were most likely responsible for the high Cd concentrations in the soils. The requirement of remediation approaches such as phytostabilization and phytoextraction by hyperaccumulator plants practices in local areas threatened by Cd [48,49]. Our findings could provide necessary information for policy makers in land use management and effectively prevent further Cd pollution.

## Supporting Information

S1 Dataset Original data of model construction. (XLS)
